# Phosphatase PP2A and microtubule-mediated pulling forces disassemble centrosomes during mitotic exit

**DOI:** 10.1242/bio.029777

**Published:** 2017-12-08

**Authors:** Stephen J. Enos, Martin Dressler, Beatriz Ferreira Gomes, Anthony A. Hyman, Jeffrey B. Woodruff

**Affiliations:** 1 Max Planck Institute of Molecular Cell Biology and Genetics, Pfotenhauerstrasse 108, 01307 Dresden, Germany; 2 Department of Cell Biology, Department of Biophysics, UT Southwestern Medical Center, Dallas, TX 75390, USA

**Keywords:** Centrosome, Disassembly, Phosphatase PP2A, SPD-5, LET-92, Pericentriolar material

## Abstract

Centrosomes are microtubule-nucleating organelles that facilitate chromosome segregation and cell division in metazoans. Centrosomes comprise centrioles that organize a micron-scale mass of protein called pericentriolar material (PCM) from which microtubules nucleate. During each cell cycle, PCM accumulates around centrioles through phosphorylation-mediated assembly of PCM scaffold proteins. During mitotic exit, PCM swiftly disassembles by an unknown mechanism. Here, we used *Caenorhabditis elegans* embryos to determine the mechanism and importance of PCM disassembly in dividing cells. We found that the phosphatase PP2A and its regulatory subunit SUR-6 (PP2A^SUR-6^), together with cortically directed microtubule pulling forces, actively disassemble PCM. In embryos depleted of these activities, ∼25% of PCM persisted from one cell cycle into the next. Purified PP2A^SUR-6^ could dephosphorylate the major PCM scaffold protein SPD-5 *in vitro*. Our data suggest that PCM disassembly occurs through a combination of dephosphorylation of PCM components and force-driven fragmentation of the PCM scaffold.

## Abstract (precis)

**Summary:** Centrosomes acquire and lose pericentriolar material during each cell cycle. We show that dephosphorylation and cortically directed forces disassemble pericentriolar material at the end of mitosis.

## INTRODUCTION

Centrosomes are micron-scale, membrane-less organelles that nucleate microtubule arrays. They are crucial for assembling and positioning the mitotic spindle, establishing membrane polarity, and asymmetric cell division. Centrosomes comprise a pair of nanometer-scale centrioles that organize a micron-scale mass of protein called pericentriolar material (PCM). PCM is required for proper centriole duplication (Dammermann et al., 2004; Loncarek et al., 2008) and determines the activity of centrosomes by serving as a concentration compartment for proteins that nucleate microtubules (Conduit et al., 2015; Woodruff et al., 2014). During each cell cycle, the PCM assembles around centrioles in preparation for mitosis, and then rapidly disassembles during mitotic exit while the centrioles persist. Post-mitotic cells often lose their PCM and centrioles altogether, suggesting a tight coupling of centrosome assembly status to cellular differentiation. In fact, centrosome disassembly is essential for female gamete formation in several organisms (Borrego-Pinto et al., 2016; Mikeladze-Dvali et al., 2012; Pimenta-Marques et al., 2016) and correlates with terminal differentiation of heart tissue in mice (Zebrowski et al., 2015). However, the importance of centrosome disassembly for mitotically dividing cells is not known. Additionally, the mechanism driving PCM disassembly is not known in any context.

PCM forms through phosphorylation-regulated assembly of long coiled-coil proteins into micron-scale scaffolds. These scaffolds then recruit client proteins, such as microtubule-stabilizing enzymes and tubulin, which are needed for centrosome function (Conduit et al., 2015; Woodruff et al., 2014). Polo kinase phosphorylation of Cdk5Rap2, Centrosomin, and SPD-5 is essential for PCM assembly in vertebrates, flies, and *C. elegans*, respectively (Conduit et al., 2010; Lee and Rhee, 2011; Woodruff et al., 2015). Furthermore, Polo kinase phosphorylation of Centrosomin and SPD-5 directly enhances their assembly into supramolecular scaffolds *in vitro* (Conduit et al., 2014; Feng et al., 2017; Woodruff et al., 2015, 2017). These results imply that removal of these phosphate moieties is important for PCM disassembly, but this idea has yet to be tested.

In this study, we set out to determine how PCM disassembles during mitotic exit in *C. elegans* embryos. We demonstrate that depletion of the PP2A phosphatase or its regulatory subunit SUR-6 slows down disassembly of the SPD-5 scaffold. Eliminating microtubule-dependent pulling forces in addition to SUR-6 depletion inhibited SPD-5 scaffold disassembly even further. We show that purified PP2A^SUR-6^ complexes dephosphorylate SPD-5 *in vitro* and that shear forces are sufficient to disrupt PCM scaffolds *in vitro*. Our results suggest that *C. elegans* PCM disassembles through dephosphorylation and microtubule-driven fragmentation of the SPD-5 scaffold.

## RESULTS

### Depletion of PP2A^SUR-6^ or microtubule-dependent pulling forces inhibits PCM disassembly *in vivo*

**Fig. 1.**
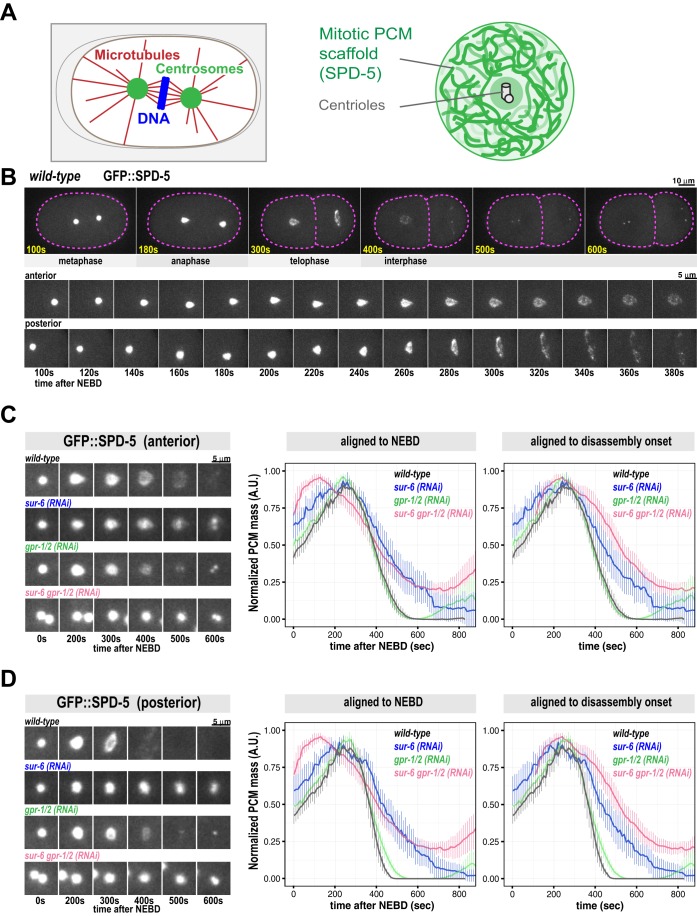
**PCM disassembly is inhibited in *sur-6(RNAi)* and *gpr-1/2(RNAi)* embryos.** (A) Diagram of mitotic spindle (left) and centrosome organization (right) in *C. elegans* embryos. (B) Confocal fluorescence images of *C. elegans* embryos expressing GFP::SPD-5, a marker for the PCM scaffold. During PCM disassembly in the 1-cell embryo, the anterior (left side) and posterior (right side) centrosomes display different morphologies. Cell outline is in magenta. Magnified images of the centrosomes are shown in the bottom panels. See also Movie 1. (C) Measurement of anterior PCM disassembly in wild-type and RNAi-treated embryos [mean with 95% confidence intervals; *n*=13 (wild-type), 9 (*sur-6(RNAi)*), 11 (*gpr-1/2 (RNAi)*)]. For *sur-6 gpr-1/2(RNAi)* (*n*=11), anterior and posterior centrosomes could not be properly distinguished; thus, the curve represents pooled data from all centrosomes. Data are normalized. See Movies 3-6 and Fig. S1B for images of the embryos. (D) Measurement of posterior PCM disassembly in wild-type and RNAi-treated embryos [mean with 95% confidence intervals; *n*=13 (wild-type), 9 (*sur-6(RNAi)*), 11 *(gpr-1/2 (RNAi)*)]. Note: for *sur-6 gpr-1/2(RNAi),* the curve from Fig. 1C is shown again for comparison (see above). Data are normalized. See Movies 3-6 and Fig. S1B for images of the embryos.

We used time-lapse microscopy to monitor PCM disassembly in *C. elegans* embryos expressing GFP-labeled SPD-5 (GFP::SPD-5), the main component of the PCM scaffold (Hamill et al., 2002; Woodruff et al., 2015, 2017) (Fig. 1A and B). Confirming previous analysis (Decker et al., 2011; Woodruff et al., 2015), PCM localized around centrioles shortly after fertilization, then grew in size as the embryo progressed toward mitosis (Movie 1). After anaphase onset, PCM expanded rapidly and then disintegrated as material simultaneously transited toward the cell cortex and dissolved (Fig. 1B). During disassembly, anterior PCM deformation was relatively isotropic, whereas posterior PCM deformation occurred primarily along the short axis of the embryo (Fig. 1B). Quantification of PCM disassembly using semi-automated tracking and segmentation revealed that PCM mass peaks ∼275 s after nuclear envelope breakdown (NEBD), corresponding to anaphase. PCM is no longer detectable 500-600 s after NEBD, corresponding to interphase of the next cell cycle; posterior PCM disassembled faster than anterior PCM (Fig. 1C and D; Movies 1 and 3). We conclude that PCM disassembly is completed in ∼4-5 min and involves fragmentation and dissolution of the SPD-5 scaffold.

PCM assembly is driven in part by PLK-1 (Polo-like Kinase) phosphorylation of SPD-5. In embryos, inhibition of PLK-1 or mutation of four PLK-1 target sites on SPD-5 prevents PCM growth (Woodruff et al., 2015; Wueseke et al., 2016)*. In vitro*, PLK-1 phosphorylation of the same four sites accelerates assembly of SPD-5 into supramolecular scaffolds (Woodruff et al., 2015). To check whether dephosphorylation of these PLK-1 sites is critical for PCM disassembly, we performed a small-scale RNAi screen against known mitotic phosphatases. RNAi-mediated depletion of the PP2A phosphatase LET-92 inhibited PCM disassembly (Fig. S1A; Movie 2). PP2A phosphatase localizes to centrosomes and connects to SPD-5 indirectly through the adapter proteins RSA-1 and RSA-2 (Schlaitz et al., 2007). Depletion of the catalytic subunit LET-92 causes pleiotropic effects such as reduced microtubule stability, mitotic spindle collapse, and increased autophagy, which could indirectly affect PCM disassembly (Lehmann et al., 2017; Schlaitz et al., 2007). PP2A phosphatases function as holoenzymes comprising an invariant catalytic and structural subunit coupled to variable regulatory subunits that determine substrate specificity (Janssens and Goris, 2001). Depletion of the conserved B55α regulatory subunit [SUR-6 in *C. elegans* (Kao et al., 2004)] by RNAi prevented complete PCM disassembly without affecting spindle size or asymmetric cell division (Fig. 1C and D; Fig. S3A; Movie 4). *sur-6* depletion also reduced the speed of PCM disassembly by 54%-65% (anterior versus posterior; see Fig. S1D for a comparison of disassembly rates). We conclude that PP2A coupled to SUR-6 (PP2A^SUR-6^) in part drives PCM disassembly.

Depletion of PP2A activity slowed down, but did not completely prevent, PCM disassembly, suggesting that additional mechanisms are required. Centrosomes are constantly under tension during anaphase due to pulling forces mediated by cortically anchored dyneins that attach to and walk along astral microtubules emanating from PCM (Grill et al., 2001; Nguyen-Ngoc et al., 2007; Severson and Bowerman, 2003). In the *C. elegans* one-cell embryo, pulling forces are ∼1.5-fold stronger in the posterior side compared to the anterior side (Grill et al., 2003). To test if microtubule-dependent pulling forces disassemble PCM, we knocked down the cortical dynein anchor GPR-1/2 by RNAi. In these embryos, PCM still disassembled, albeit without the dramatic expansion in size seen in wild-type embryos (Fig. 1C and D; Movie 5) (Severson and Bowerman, 2003). The rate of disassembly was reduced ∼27% for posterior centrosomes in *gpr-1/2(RNAi)* embryos compared to wild-type embryos; conversely, we did not observe any change in the rate of anterior centrosome disassembly (Fig. 1D; Fig. S1D). Thus, elimination of microtubule-pulling forces has a minor effect on disassembly of the posterior centrosome. Interestingly, PCM assembly was detectable much sooner in the subsequent cell cycle in *grp-1/2(RNAi)* embryos compared to wild-type embryos for both anterior and posterior centrosomes (Fig. 1C and D). These results suggest that an active but barely detectable layer of PCM persists after mitotic exit in *grp-1/2(RNAi)* embryos; this layer which could prematurely seed PCM accumulation in the next cell cycle.

Combinatorial depletion of GPR-1/2 and SUR-6 resulted in a more severe PCM disassembly phenotype: PCM disassembly was 59%-72% slower than wild-type (anterior versus posterior) and ∼25% of the original PCM mass persisted into the next cell cycle (Fig. 1C and D; Fig. S1D; Movie 6). We also noticed additional defects in *sur-6 gpr-1/2(RNAi)* embryos, such as mitotic spindle collapse and altered cell cycle progression. In particular, the time between NEBD and disassembly onset was shorter in *sur-6 gpr-1/2(RNAi)* embryos compared to *sur-6(RNAi)*, *gpr-1/2(RNAi)*, or wild-type embryos. This could be due to the fact that pronuclear contact is delayed in *sur-6 gpr-1/2(RNAi)* embryos (Fig. S2A,B), which might allow the centrosomes to advance in their cycle, thereby accelerating the onset of disassembly relative to NEBD. Thus, for comparison purposes, we display the PCM disassembly curves aligned by NEBD and by disassembly onset (Fig. 1C,D). We conclude that PP2A^SUR-6^ and microtubule pulling forces cooperate to disassemble PCM.

In early *C. elegans* embryos PCM grows until reaching a stereotyped upper limit (Decker et al., 2011). We wondered if PCM disassembly mechanisms help set this upper limit. As expected, *gpr-1/2* depletion slightly increased PCM mass in anaphase. Unexpectedly, *sur-6* depletion actually decreased PCM mass (see Fig. S1C for non-normalized data). PCM mass in anaphase in *sur-6 gpr-1/2(RNAi)* embryos was slightly lower than in wild-type embryos (Fig. S1C). These results suggest that GPR-1/2 opposes PCM assembly and that SUR-6 affects both PCM growth and disassembly. At this time, we do not know how SUR-6 could affect PCM assembly.

### PP2A^SUR-6^ dephosphorylates a key PLK-1 site on SPD-5

**Fig. 2.**
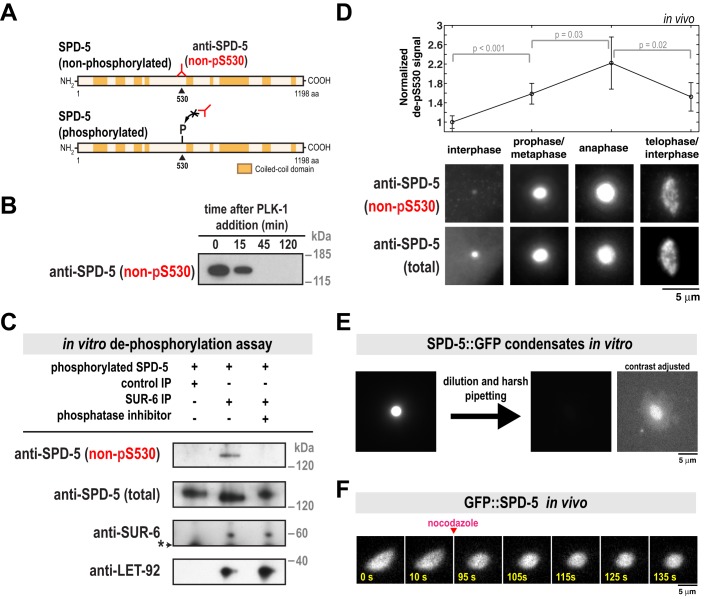
**PP2A^SUR-6^ dephosphorylates SPD-5 and shear stresses distort and dissolve SPD-5 assemblies *in vitro*.** (A) SPD-5 domain architecture and location of the serine 530 phospho-epitope. The non-pS530 antibody recognizes serine 530 only when not phosphorylated. (B) 200 nM of purified SPD-5 was incubated with 200 nM PLK-1+0.2 mM ATP. The reaction was analyzed at various time points by western blot using the non-pS530 antibody. (C) *In vitro* dephosphorylation assay. Control beads affixed to CDC-37 antibody or beads affixed to SUR-6 antibody were incubated in *C. elegans* embryo extract, then washed and resuspended in buffer. SPD-5 that was pre-phosphorylated *in vitro* by PLK-1 was then added and incubated for 90 min at 23°C and analyzed by western blot. Calyculin A was used as the phosphatase inhibitor (lane 3). The asterisk indicates a non-specific band. (D) Immunofluorescence was used to monitor relative changes in SPD-5 phosphorylation over the cell cycle. *C. elegans* embryos were co-stained with non-pS530 antibody and a general polyclonal SPD-5 antibody (total SPD-5). To control for changes in non-pS530 signal due to PCM growth, the ratio of the two antibody signals (non-pS530/total SPD-5) was measured for four cell cycle stages. These values were then normalized against the interphase value (mean with 95% confidence intervals; *n*=19 interphase, 48 prophase/metaphase, 17 anaphase, and 33 telophase centrosomes; *P*-values are from an unpaired *t*-test). (E) SPD-5 condensates were formed by incubating 500 nM SPD-5::GFP in 9% PEG-3350 for 27 min at 23°C (left), then diluted 1:10 into a 0% PEG solution and pipetted harshly (right). Dilution of SPD-5 condensates into PEG-free solution is necessary to prevent their reformation. SPD-5 condensates become more resistant to dilution as they age, thus the observed disruption and dissolution is due to pipetting (Woodruff et al., 2017; and unpublished data). The two images on the right are the same, except the contrast has been increased in the far right image to show the disrupted SPD-5 condensate. (F) Semi-permeable *perm-1(RNAi)* embryos expressing GFP::SPD-5 were treated with 20 µg/ml nocodazole once PCM deformation was apparent (∼250-300 s after NEBD). PCM relaxes to a spherical shape after microtubules are depolymerized (*n*=4 embryos).

SPD-5 assembly is accelerated by PLK-1 phosphorylation at four central serine residues within SPD-5 (S530, S627, S653, S658) (Woodruff et al., 2015). To determine if PP2A^SUR-6^ dephosphorylates these residues, we generated a monoclonal antibody that specifically recognizes S530 only in the non-phosphorylated state (Fig. 2A). Western blot analysis showed that this antibody recognizes purified non-phosphorylated SPD-5 *in vitro*. As expected, the antibody signal declined when SPD-5 was phosphorylated by purified PLK-1 (Fig. 2B). We refer to this antibody hereafter as ‘non-pS530’.

We next performed an *in vitro* dephosphorylation assay to test if PP2A^SUR-6^ can directly dephosphorylate SPD-5 at S530. We affixed SUR-6 antibodies to beads to isolate PP2A^SUR-6^ complexes from *C. elegans* embryo extracts. Western blot analysis confirmed that these complexes contained the regulatory subunit SUR-6 and the catalytic subunit LET-92 (Fig. 2C). The PP2A^SUR-6^ beads were then resuspended in buffer that contained purified SPD-5 that had been pre-phosphorylated *in vitro* by PLK-1. As shown in Fig. 2C, SPD-5 was dephosphorylated only in the presence of active PP2A^SUR-6^. Non-pS530 signal was not present when control beads were used or if the PP2A inhibitor Calyculin A was included. These results suggest that PP2A^SUR-6^ drives PCM disassembly by dephosphorylating SPD-5.

We then performed dual color immunofluorescence with the non-pS530 antibody and a general SPD-5 antibody to assess relative changes in SPD-5 dephosphorylation during the cell cycle. The non-pS530 antibody localized to centrosomes in all cell cycle stages, corroborating our previous observation that a phospho-mutant version of SPD-5 (GFP::SPD-5^4A^) localizes to centrosomes when wild-type SPD-5 is present (Wueseke et al., 2016). Quantification of non-pS530 antibody signal showed that it was low during interphase, increased during metaphase, then peaked during anaphase, coincident with disassembly onset (Fig. 2D). This result suggests that PCM disassembly is associated with dephosphorylation of SPD-5. Later in telophase and the subsequent interphase, non-pS530 signal decreased (Fig. 2D). It is possible that dephosphorylated SPD-5 is weakly associated with the PCM and removed faster than phosphorylated SPD-5, as predicted by our previous model (Wueseke et al., 2016).

### Forces disassemble the SPD-5 scaffold *in vitro* and distort PCM *in vivo*

Our analysis of *grp-1/2(RNAi)* embryos suggested that cortically directed pulling of microtubules assists PP2A^SUR-6^ in driving PCM disassembly. To test if force is sufficient to disassemble the PCM scaffold, we applied shear stress to SPD-5 assemblies *in vitro*. Purified SPD-5 forms PCM-like scaffolds that concentrate PCM client proteins like PLK-1, microtubule stabilizing enzymes, and tubulin. Depending on macromolecular crowding conditions, SPD-5 assembles either into dense spherical condensates or irregular networks (Woodruff et al., 2015, 2017). Application of shear force by harsh pipetting completely disassembled the less dense SPD-5 networks (Fig. S2) and partially disassembled the denser SPD-5 condensates (Fig. 2E). After harsh pipetting, some SPD-5 condensates lost their spherical morphology (Fig. 2E). To validate these observations *in vivo*, we performed acute disruption of microtubule-dependent forces during anaphase by treating permeable embryos with 20 µg/ml nocodazole. After nocodazole application, the normally elongating PCM scaffold relaxed to a spherical shape (Fig. 2F). Taken together, these results suggest that cortically directed forces are integral in fragmenting and disassembling the PCM scaffold.

### *sur-6 gpr-1/2(RNAi)* embryos display abnormal centrosome accumulation and improper cell divisions

**Fig. 3.**
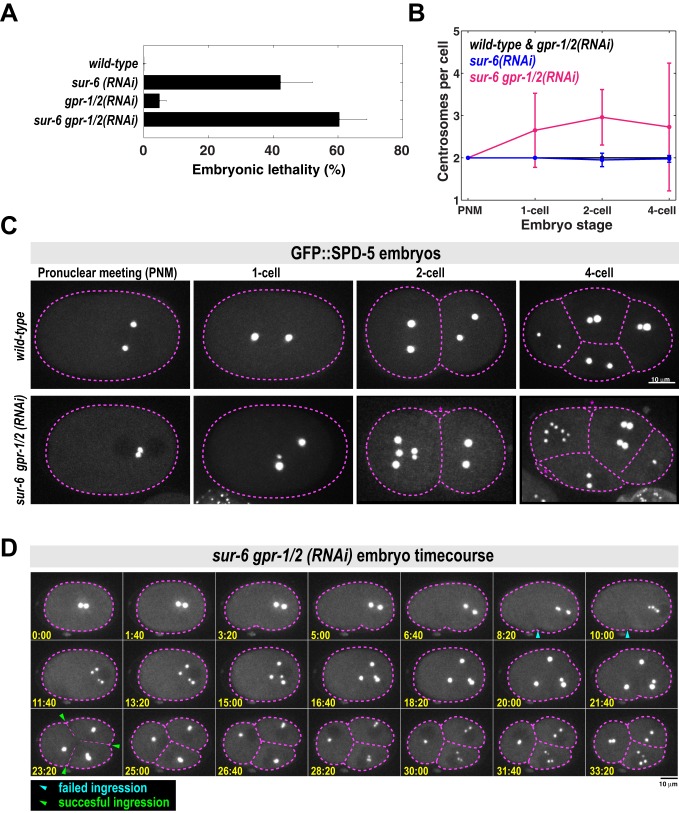
***sur-6 gpr-1/2 (RNAi)* embryos display abnormal centrosome numbers and cell divisions.** (A) Analysis of embryonic lethality in various partial RNAi conditions. For *sur-6(RNAi)*, L4 worms were grown on *sur-6* feeding plates for 24 h at 23°C. For *gpr-1/2(RNAi)*, L4 worms were grown on control feeding plates for 16 h at 23°C, then transferred to *grp-1/2* feeding plates for 8 h at 23°C. For *sur-6 gpr-1/2(RNAi)*, L4 worms were grown on *sur-6* feeding plates for 16 h at 23°C, then transferred to *sur-6 gpr-1/2* feeding plates for an additional 8 h at 23°C (*n*=8 mothers per condition and >50 F1 embryos per mother). (B) Number of centrosomes per cell in wild-type, *sur-6(RNAi), gpr-1/2(RNAi)* and *sur-6 gpr-1/2(RNAi)* embryos (mean±s.d.; *n*=10 embryos in each condition). All embryos contained only two centrosomes during pronuclear meeting (PNM), which occurs shortly after fertilization. 3-cell embryos were not counted, as they are transient in the wild-type condition and do not have visible centrosomes during that brief time. (C) Representative images from B. Cell outline is in magenta. Blebs were visible in the *sur-6 gpr-1/2 (RNAi)* embryos, but were not counted as cells (magenta asterisks). (D) Time-lapse imaging of a *sur-6 gpr-1/2(RNAi)* embryo expressing GFP::SPD-5. Blue arrowheads indicate abortive cytokinetic furrow ingression. Green arrowheads indicate successful furrow ingression. See also Movie 6.

PCM assembles and disassembles during each mitotic cell cycle in dividing metazoan cells. However, it is not clear why PCM must disassemble instead of persisting through each cell cycle like other organelles, such as mitochondria. We thus tested the impact of inhibiting PCM disassembly on embryo viability. *sur-6* null embryos [*sur-6(sv30)*] or embryos treated with *gpr-1/2(RNAi)* for 24 h at 23°C resulted in near 100% lethality (Gotta et al., 2003; Kao et al., 2004). When we reduced the strength of RNAi treatment, we observed a weak genetic interaction between *sur-6* and *gpr-1/2* (see Fig. 3A for details)*.* Under these conditions, lethality of F1 embryos was 0% in wild-type, 40% in *sur-6(RNAi)*, 5% in *gpr-1/2(RNAi)* and 60% in *sur-6 gpr-1/2(RNAi)* worms (Fig. 3A). It is possible that this synthetic lethality results from inhibition of PCM disassembly. However, SUR-6 and GPR-1/2 are also known to regulate centriole duplication and spindle positioning, respectively (Gotta et al., 2003; Kao et al., 2004; Song et al., 2011). Disruption of these processes could contribute to the embryonic lethal phenotype. We therefore analyzed the earliest cell divisions in *C. elegans* embryos, which are known to be largely unaffected by single RNAi depletion of *sur-6* and *gpr-1/2* (Kao et al., 2004; Nguyen-Ngoc et al., 2007; Song et al., 2011).

We noticed severe mutant phenotypes in *sur-6 gpr-1/2(RNAi)* embryos that were not present in *sur-6(RNAi)* or *gpr-1/2(RNAi)* embryos. For example, metaphase spindles were noticeably shorter in *sur-6 gpr-1/2(RNAi)* embryos (Fig. S3A). Furthermore, wild-type, *sur-6(RNAi)*, and *gpr-1/2(RNAi)* embryos predominantly contained only two centrosomes per cell. A few *sur-6(RNAi)* embryos had one centrosome per cell (5% and 2.5% of cells in 2-cell and 4-cell embryos, respectively), as expected (Kitagawa et al., 2011). On the other hand, *sur-6 gpr-1/2(RNAi)* embryos often contained >2 centrosomes; sometimes up to 7 centrosomes per cell could be seen by the 4-cell stage (Fig. 3B and C).

We then followed embryo development with time-lapse microscopy to understand how abnormal centrosome numbers arise in *sur-6 gpr-1/2(RNAi)* embryos (Fig. 3D; Movie 3). All *sur-6 gpr-1/2(RNAi)* embryos contained only two centrosomes after fertilization (*n*=16 embryos), indicating that failed meiotic divisions in the sperm were not responsible for the centrosome accumulation phenotype (Fig. 3C and D; see pronuclear meeting stage). At the end of the first cell cycle, centrosomes separated, but cytokinetic furrow ingression failed in 15 out of 16 embryos. These centrosomes maintained PCM from the previous cell cycle but still managed to split into two new centrosomes and retain their spherical shape. They then accumulated PCM and formed new spindles, indicating cell cycle progression into the next mitosis (16/16 embryos). After the second mitotic cycle, cytokinesis occurred in all embryos. We never observed centrosome over-duplication within one cell cycle. Thus, we conclude that extra centrosomes accumulate due to failed cytokinesis combined with a normal centrosome duplication cycle. We sometimes saw odd numbers of centrosomes per cell due to incomplete centrosome splitting (Fig. 3C; Fig. S3B). Because these phenotypes appear only in the double RNAi condition, they likely arise from the genetic interaction between *sur-6* and *gpr-1/2*. Since we have shown that SUR-6 and GPR-1/2 cooperate during PCM disassembly, it is possible that the mutant phenotypes are a consequence of failed PCM disassembly. However, we cannot discount the possibility that SUR-6 and GPR-1/2 cooperate in an unknown manner to regulate spindle assembly or cytokinesis, independent of their effects on PCM disassembly. Future work using acute chemical inhibition of PP2A and microtubule depolymerization during anaphase is required to distinguish between these possibilities.

## DISCUSSION

In this study we have shown that PP2A phosphatase and cortically directed pulling forces are required for disassembly of PCM, the outer layer of centrosomes responsible for nucleating microtubules. Our results lead us to propose the following model for PCM assembly, maturation, and disassembly in *C. elegans* embryos. Prior to mitosis, PCM forms through self-assembly of the scaffold protein SPD-5 into micron-scale spherical condensates that then concentrate PCM client proteins, such as tubulin, needed for microtubule aster nucleation. SPD-5 scaffold formation is accelerated by the nucleator SPD-2 and PLK-1 phosphorylation of SPD-5. During mitotic exit, PP2A (LET-92 in *C. elegans*) coupled to its regulatory subunit B55α (SUR-6 in *C. elegans*) drives PCM disassembly by opposing PLK-1 and dephosphorylating the scaffold protein SPD-5. Simultaneously, the PCM scaffold weakens and outward-pulling forces mediated by microtubules fragment the SPD-5 scaffold.

How could dephosphorylation of SPD-5 promote PCM disassembly? So far, we have not observed disassembly of SPD-5 condensates in the presence of phosphatase *in vitro* (J.B.W. unpublished data). This result suggests that once the SPD-5 scaffold is formed, dephosphorylation is not sufficient to destabilize it. This is possible considering that PLK-1 phosphorylation is not strictly required for SPD-5 assembly but rather affects the rate of assembly *in vitro* (Wueseke et al., 2016). Furthermore, the mature SPD-5 scaffold is remarkably stable and displays little to no turnover *in vivo* and *in vitro*, regardless of phosphorylation status (Laos et al., 2015; Woodruff et al., 2017; Wueseke et al., 2016). It is also unlikely that dephosphorylation promotes SPD-5 degradation, as SPD-5 levels are relatively constant throughout the cell cycle (Wueseke et al., 2014). Instead, we propose that dephosphorylation of SPD-5 impedes its reassembly after dissociating from the PCM. Dephosphorylation of SPD-5 may also impact its stability within the PCM, perhaps by decreasing the affinity of SPD-5 for itself.

SPD-5 is likely not the only protein that is dephosphorylated during mitotic exit. It is possible that PP2A^SUR-6^ also dephosphorylates regulators of PCM assembly such as SPD-2, PLK-1, and Aurora A kinase; each of these proteins is activated in part by phosphorylation (Decker et al., 2011; Littlepage et al., 2002; Qian et al., 1998). In the future, it will be important to analyze the role of dephosphorylation and departure of SPD-2, PLK-1, and Aurora A kinase for PCM disassembly.

How can force-induced fragmentation drive PCM disassembly? Our data show that shear stress directly destabilizes SPD-5 assemblies *in vitro*. However, elimination of microtubule-pulling forces only slightly inhibits PCM disassembly *in vivo*. This could be a difference in force magnitude and the type of force applied, as pipetting (*in vitro*) would cause shear strain, while pulling (*in vivo*) would cause linear strain. It is also possible that PCM fragmentation increases the exposed surface of the PCM scaffold, making it more accessible to disassembly enzymes that would otherwise be excluded. This would not apply to PP2A, which concentrates at centrosomes (Schlaitz et al., 2007) and has a clear effect on PCM disassembly even when pulling forces are eliminated (Fig. 1C). However, this principle could apply to as-of-yet unidentified disassembly mechanisms. It is likely that additional mechanisms drive PCM disassembly, since centrosomes still lost 75% of their peak PCM mass in *sur-6 gpr-1/2(RNAi)* embryos. Of course, we acknowledge that RNAi-knockdown of *sur-6* and *gpr-1/2* is incomplete (Gotta et al., 2003; Song et al., 2011); complete knockout of these genes or their activity using small molecule inhibitors would likely worsen the disassembly phenotype.

We hypothesize that the combination of PP2A^B55α^ and microtubule-mediated pulling forces disassemble PCM in other species. Polo kinase phosphorylation of coiled-coil scaffold proteins, like Centrosomin and Pericentrin, is required for PCM assembly in flies and humans (Conduit et al., 2014; Lee and Rhee, 2011), suggesting that dephosphorylation would be required for disassembly. Supporting this idea, in fly embryos, Centrosomin appears to be dephosphorylated at the centrosomal periphery where disassembly occurs (Feng et al., 2017). PP2A and its regulator subunit B55α are conserved in flies, but it is not known if PP2A^B55α^ dephosphorylates Centrosomin. Microtubule-mediated pulling forces position the mitotic spindle in most somatic eukaryotic cell types (McNally, 2013), and those forces normally deform and eject PCM in fly embryos (Conduit et al., 2014; Megraw et al., 2002). It remains to be seen if such forces are required for PCM disassembly in flies and other eukaryotes.

## MATERIALS AND METHODS

### Worm strain maintenance and RNA interference

*C. elegans* worm strains were grown on NGM plates at 16-23°C, following standard protocols (www.wormbook.org). We used one strain with the following genotype:

OD847: unc-119(ed9) III; ltSi202[pVV103/ pOD1021; Pspd-2::GFP::SPD-5 RNAi-resistant; cb-unc-119(+)]II

RNA interference was performed by feeding. For nocodazole treatment of embryos, L4 worms were grown on *perm-1(RNAi)* feeding plates at 20°C for 16-18 h, then dissected in an open imaging chamber filled with osmotic support medium (Carvalho et al., 2011; Wueseke et al., 2016) and 20 µg/ml nocodazole (Sigma). For *sur-6* and *gpr-1/2(RNAi)* treatment, L4 worms were grown on their given plates at 23°C for 24-28 h, then dissected and imaged following standard protocols.

### Imaging

For live embryo imaging, we used an inverted Olympus IX81 microscope with a Yokogawa spinning-disk confocal head (CSU-X1), a 60×1.2 NA Plan Apochromat water objective, and an iXon EM+DU-897 BV back illuminated EMCCD camera (Andor Technologies, Belfast, UK). For analysis of PCM disassembly *in vivo*, we generated 36×0.5 µm Z-stacks every 10 s using 50 ms exposure and 8% laser intensity (4.5 mW; 488 nm laser). *In vitro* SPD-5 condensates and fixed embryos were visualized with an inverted Olympus IX71 microscope using 60×1.42 NA or 100×1.4 NA Plan Apochromat oil objectives, CoolSNAP HQ camera (Photometrics Tuscon, AZ, USA), and DeltaVision control unit (GE Healthcare, Salt Lake City, UT, USA).

### Assembly of SPD-5 condensates *in vitro*

SPD-5 condensates were formed by adding concentrated SPD-5::GFP to condensate buffer (25 mM HEPES, pH 7.4, 150 mM KCl) containing polyethylene glycol (molecular weight 3350 Da) and fresh 0.5 mM DTT. See Woodruff et al. (2015) for details on SPD-5 purification.

### *In vitro* kinase assay

For the experiment in Fig. 2B, 200 nM SPD-5::GFP, 200 nM PLK-1::6xHis, and 0.1 mg/ml ovalbumin were incubated in kinase buffer (20 mM Tris, pH 7.4, 150 mM KCl, 10 mM MgCl_2_, 0.2 mM ATP, 1 mM DTT) for 1 h at 23°C.

### Centrosome tracking and quantification

For all centrosome disassembly measurements, acquired stacks were analyzed by a custom-made FIJI macro (see Fig. S4).

### Movie generation and alignment

First, Z stacks were collapsed into SUM projections at each time point and then combined to make time-lapse movies. Each movie was then split into two stacks to isolate the anterior and posterior centrosomes. The frame corresponding to nuclear envelope breakdown (NEBD) was identified, and only the frames after NEBD were processed.

### Determination of background and threshold

On the first frame, a Gaussian blur was applied (sigma=1) and the maximum intensity pixel was identified, which represents the center of the centrosome. A band-shaped region was created around the maximum intensity pixel with an inner circle that encompasses the largest extent of PCM signal (radius=7 pixels) and an outer circle (radius=10 pixels). Background mean intensity (mean_bg_) and standard deviation (stdev_bg_) were then calculated from the area between the two rings.

### Segmentation and measurement of centrosomes

Centrosomes were segmented automatically by creating a region of interest (ROI) using the following threshold: mean_bg_+3*stdev_bg_. This threshold was applied to the remaining frames. The integrated signal intensity (a proxy for PCM mass) bounded by the ROI was calculated by: (mean_ROI_-threshold)*area_ROI_. The data were normalized by dividing the integrated signal intensity of each frame by the highest value of integrated signal intensity of the stack. Since we were not able to separate the two centrosomes in the double RNAi strain, the image segmentation was done following the same method outlined above, except in two steps: the band-shaped region was created with a bigger radius and the integrated signal intensity measurements were divided by two.

### Immunofluorescence

Embryos were fixed in methanol and frozen in liquid N2, as previously described (Hamill et al., 2002), then stained with 1:2000 anti-SPD-5 (non-pS530; mouse; BX23 clone) and 1:5000 anti-SPD-5 (total; rabbit; 758 clone which recognizes a.a. 1053-1198). 1:400 goat anti-rabbit-alexa488 and 1:400 goat anti-mouse-alexa594 (Life Technologies) were used as secondaries.

### *In vitro* dephosphorylation assay and western blotting

Worms were harvested in IP buffer [1× PBS plus 100 mM KCl, 1 mM EGTA, 1 mM MgCl_2_, 1% CHAPS, and 1× Complete Protease Inhibitor cocktail (Roche)] and snap-frozen in liquid nitrogen. Frozen worm pellets were turned into powder using a Retsch MM301 mill. Worm lysate was prepared by resuspending worm powder in 1.5 ml IP buffer per gram powder. Lysate was cleared by centrifuging for 10 min at 10,000 ***g*** at 4°C. The cleared lysate was again centrifuged for 10 min at 16,000 ***g*** at 4°C. Immunoprecipitation was carried out using Dynabeads Protein G kit (Life Technologies) and anti-SUR-6 and anti-CDC-37 antibodies. Instead of eluting the protein after the immunoprecipitation, the buffer was exchanged for phosphatase buffer (40 mM Tris pH 8.4, 34 mM MgCl_2_, 4 mM EDTA, 2 mM DTT, 0.05 mg/ml BSA). SUR-6/LET-92 bound to beads was used to dephosphorylate recombinant SPD-5 phosphorylated *in vitro* using recombinant PLK-1 (see *in vitro* kinase assay; the reaction was passed over a Ni-NTA column to remove PLK-1::6xHis). Dephosphorylation was carried out at room temperature for 1.5 h in phosphatase buffer. Aliquots of the reactions were separated on 4-12% NuPAGE gradient gels (Life Technologies). Proteins were transferred onto nitrocellulose membrane using an iBlot device (Life Technologies). Membranes were blocked using 3% BSA in TBST containing 0.1% Tween-20 and probed using antibodies against total SPD-5 (1:5000; 758 clone, in-house), SPD-5 which is not phosphorylated at S530 (1:1000; BX23 clone, in-house), SUR-6 (1:500, gift from K. O'Connell, NIH) and PP2A catalytic subunit (1:2000; BD Biosciences). Secondary antibodies were HRP-conjugated goat anti-rabbit and goat anti-mouse (Bio-Rad, 1:30,000). Detection was carried out using SuperSignal ECL reagent (Bio-Rad).

## Supplementary Material

Supplementary information
