# Supplementary material for: Phosphatase PP2A and microtubule-mediated pulling forces disassemble centrosomes during mitotic exit
Source: Biol Open. 2017 Dec 8;7(1):bio029777. doi: 10.1242/bio.029777 (PMC5829501; doi:10.1242/bio.029777)
Supplement: Supplementary information [file biolopen-7-029777-s1.pdf]

Biology Open (2018): doi:10.1242/bio.029777: Supplementary information

SUPPLEMENTAL FIGURES

Enos et al. Figure S1

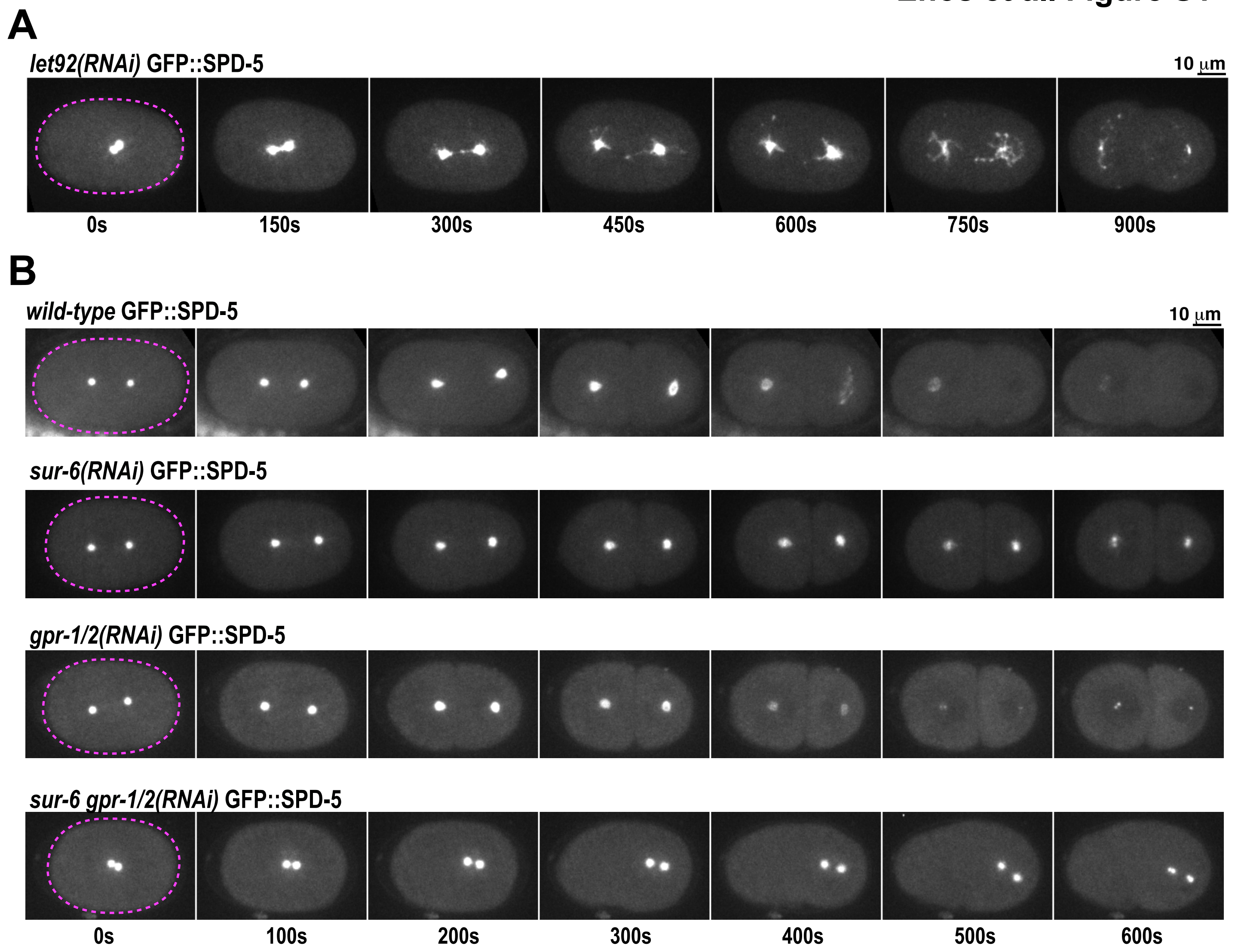

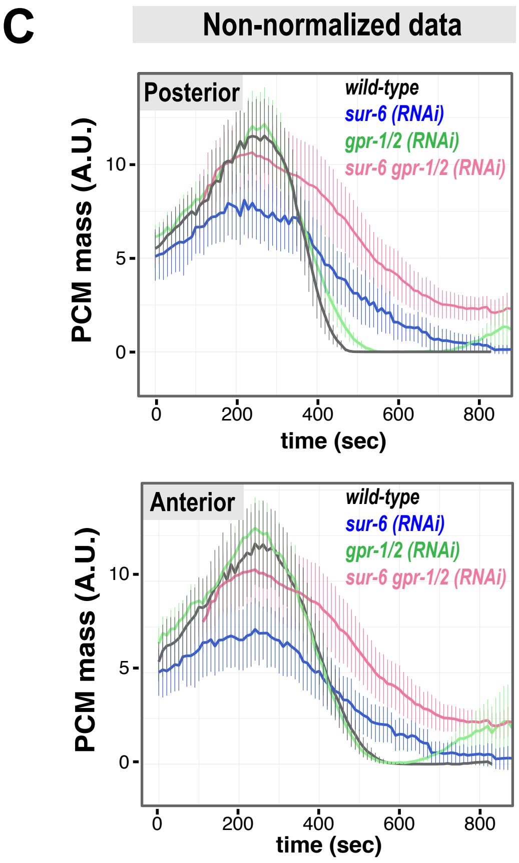

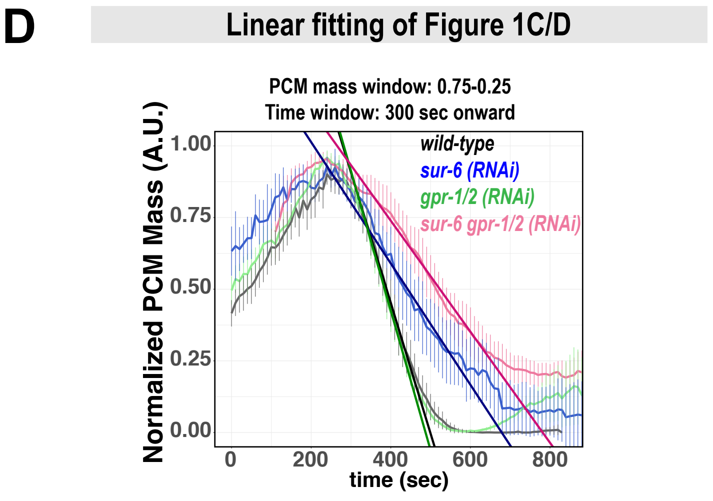

PCM disassembly rates (A.U./sec)

	anterior	posterior
wild-type	-0.0046	-0.0068
sur-6 (RNAi)	-0.0021	-0.0024
gpr-1/2 (RNAi)	-0.0049	-0.0050
sur-6 gpr-1/2 (RNAi)	-0.0019	-0.0019

Biology Open • Supplementary information

Figure S1. PCM disassembly defects in various RNAi conditions.

A. Confocal images of a *C. elegans* embryo expressing GFP::SPD-5 after *let-92* was depleted by RNAi (24 hr at 23° C). See also Movie S2.
B. Images of the entire embryos from the analysis in Figure 1C and 1D.
C. Non-normalized quantification of PCM mass from Figure 1C and 1D. Curves are aligned by peak PCM mass (mean with 95% confidence intervals).
D. PCM disassembly rates calculated by fitting the linear region of the curves in Figure 1C and 1D (linear region: window between 0.75 and 0.25 PCM mass during disassembly, corresponding to 300 s post-NEBD). An example of the fitting is shown in the plot on the right.

Enos et al. Figure S2

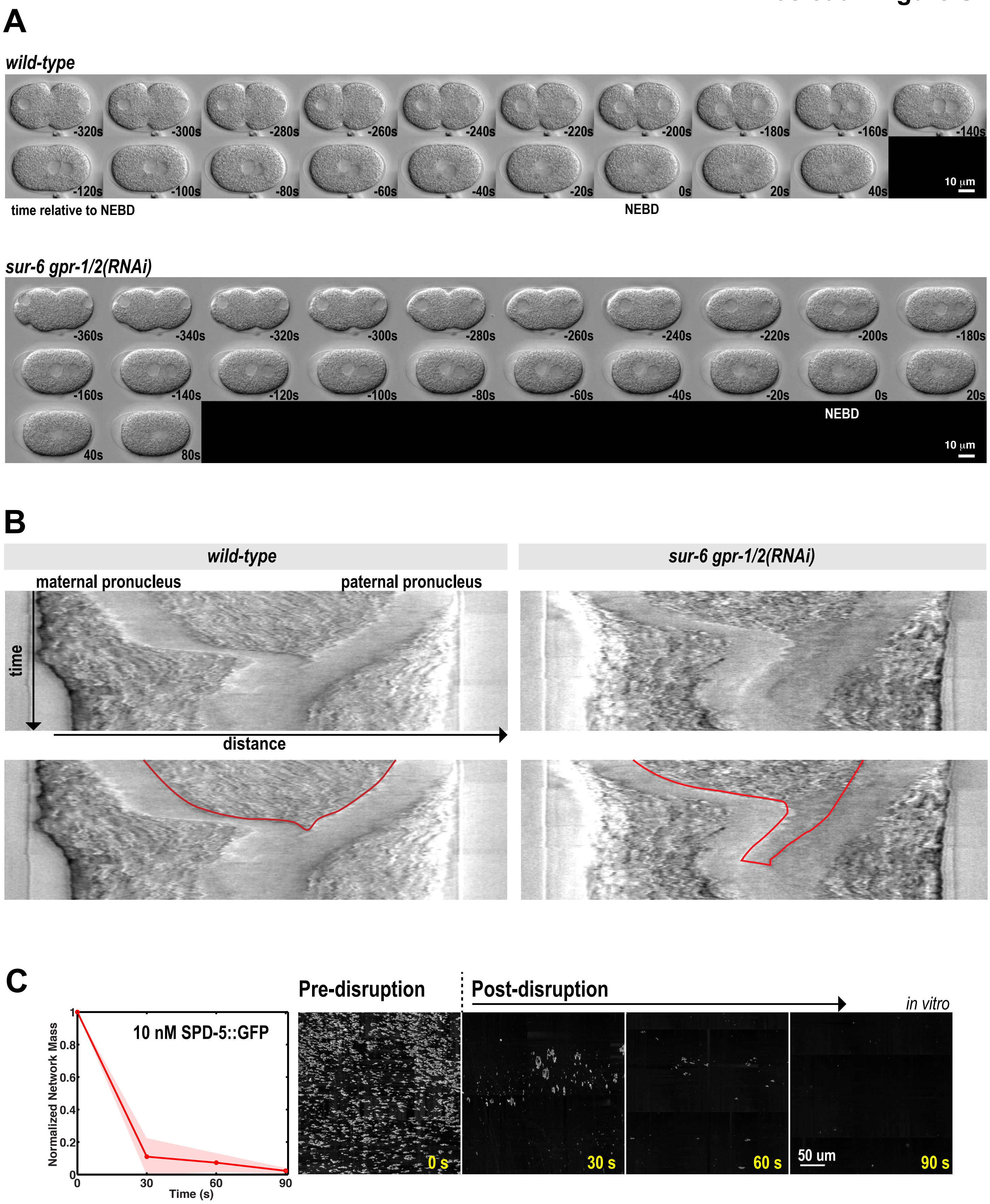

Figure S2. Analysis of pronuclear meeting *in vivo* and stability of SPD-5 networks in vitro.

A. DIC images showing pronuclear migration, pronuclear meeting, and nuclear envelope breakdown (NEBD) in 1-cell *C. elegans* embryos.

B. Kymographs of the images in (A). A slice was taken through the middle (long axis) of the embryo at each time point. The anterior is on the left and posterior on the right. In the bottom images the red line marks the inward-facing boundary of each pronucleus during migration. In *sur-6 gpr-1/2(RNAi)* embryos, the pronuclei migrate properly but halt before their membranes make contact (n = 10). This delay in contact, which delays NEBD, could potentially explain why the timing of centrosome disassembly is altered in *sur-6 gpr-1/2(RNAi)* embryos (see figure 1).

C. After 1 hr at 23° C, 10 nM SPD-5::GFP assembled into micron-scale networks. The sample was then pipetted 10 times and network mass (integrated fluorescence) was calculated at different time points as described in (Woodruff et al., 2015). The red line represents the mean, the shaded area represents the 95% confidence interval (n = 9 experiments). Networks assembled from 28 nM SPD-5::GFP also dissolved after harsh pipetting (see (Woodruff et al., 2015), quantification not shown).

Biology Open • Supplementary information

Enos et al. Figure S3

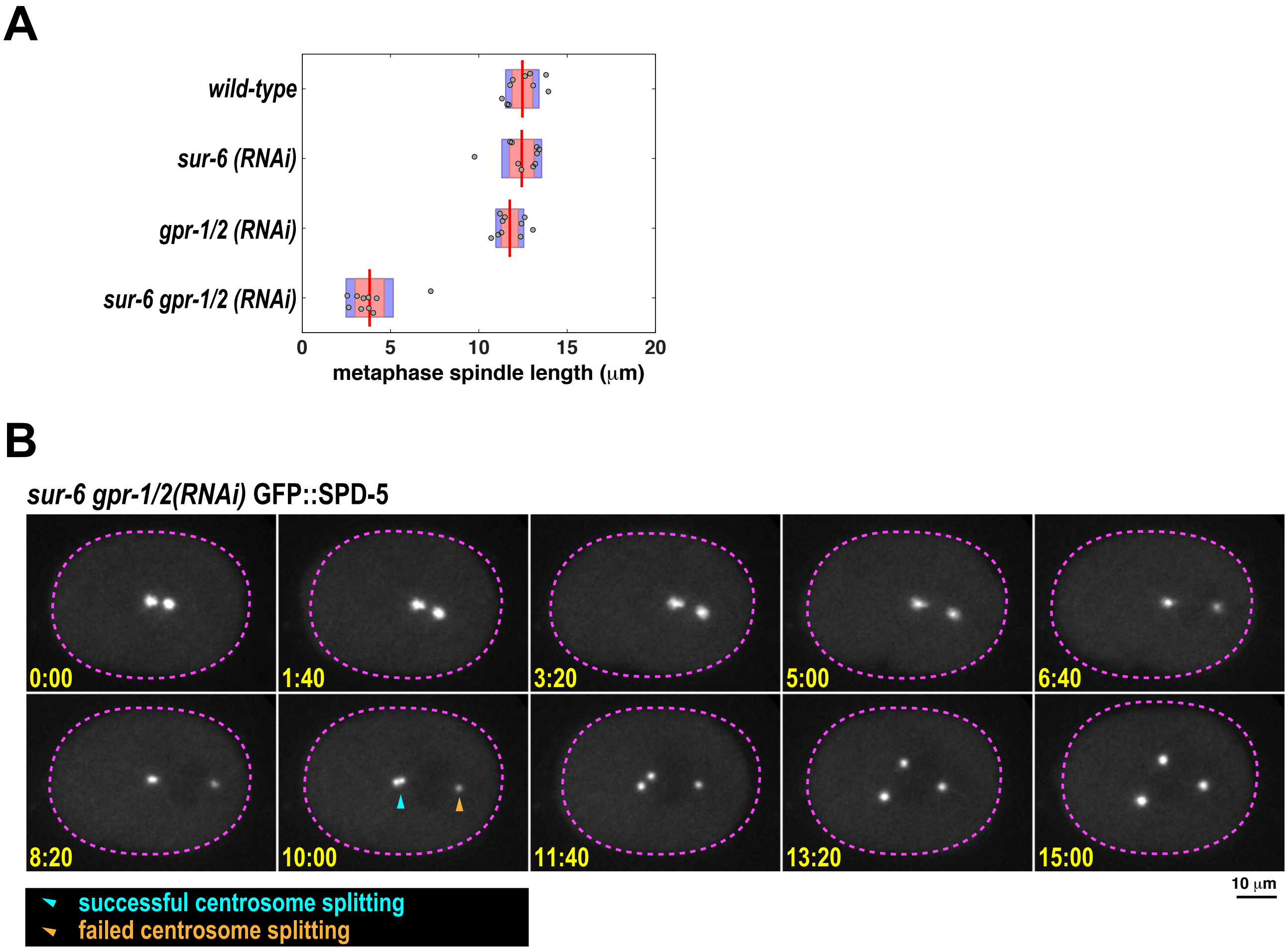

Figure S3. Analysis of metaphase spindle length and centrosome splitting.

A. Metaphase spindle length in 1-cell embryos from Figure 1C and 1D. Plot shows means (red bars), 95% confidence intervals (red shaded areas), and S.D. (blue shaded areas; n = 10 spindles per condition).

B. Example of a sur-6 gpr-1/2(RNAi) embryo where one centrosome fails to split (orange arrowhead) after onset of PCM disassembly.

Biology Open (2018): doi:10.1242/bio.029777: Supplementary information

Enos et al. Figure S4

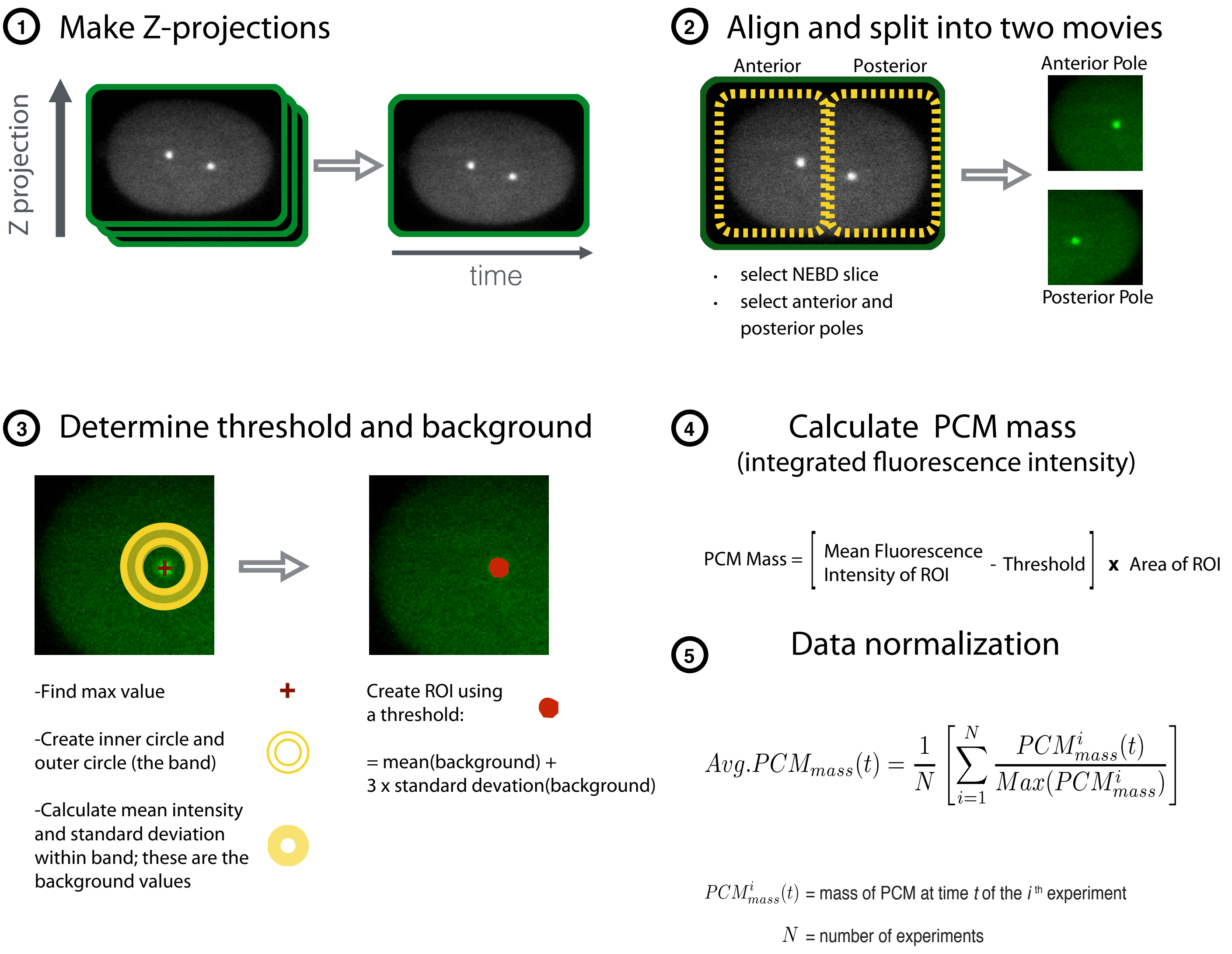

Figure S4. Outline of PCM disassembly quantification. See methods for a detailed explanation.

Biology Open • Supplementary information

SUPPLEMENTAL MOVIES

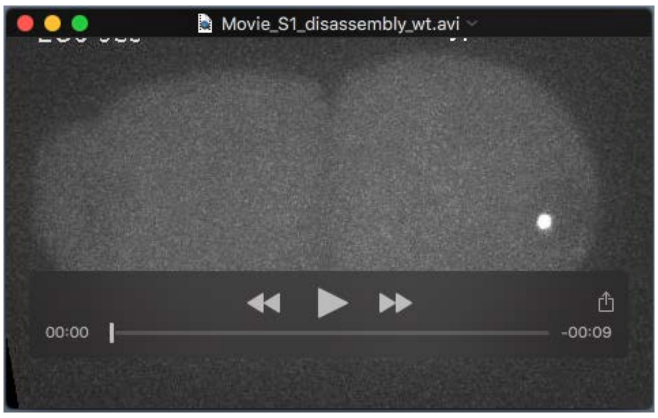

Movie S1. PCM assembly and disassembly in a one-cell wild-type embryo expressing GFP::SPD-5. Time is relative to nuclear envelope breakdown in the first cell cycle.

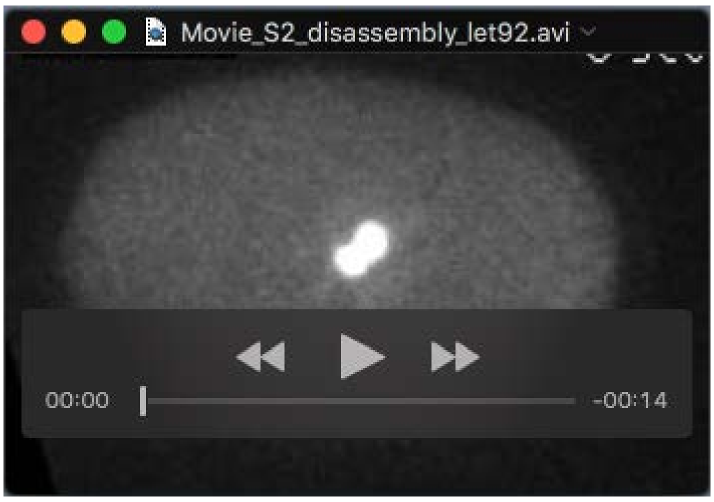

Movie S2. PCM disassembly in a *let-92(RNAi)* embryo expressing GFP::SPD-5.

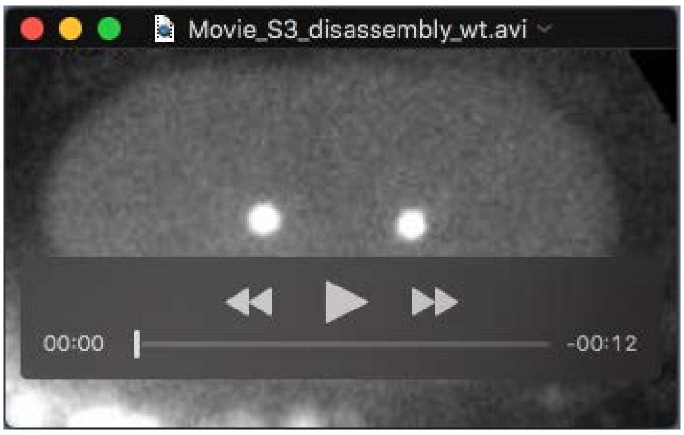

Movie S3. PCM disassembly in a wild-type embryo expressing GFP::SPD-5.

Biology Open • Supplementary information

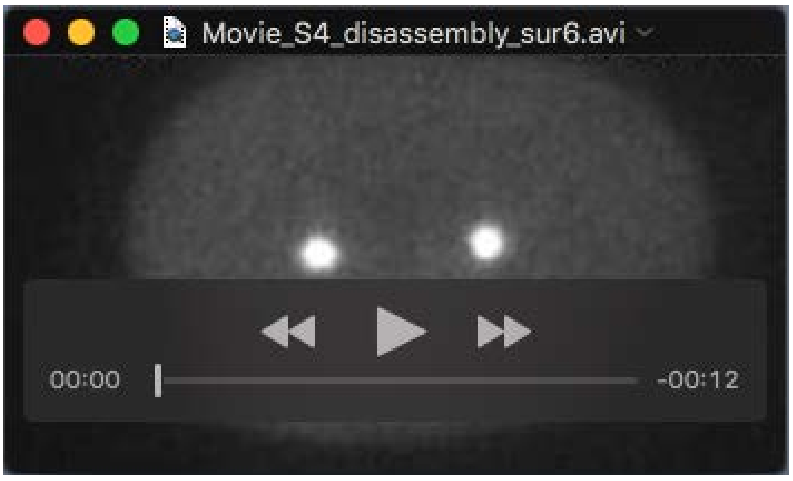

Movie S4. PCM disassembly in a *sur-6(RNAi)* embryo expressing GFP::SPD-5.

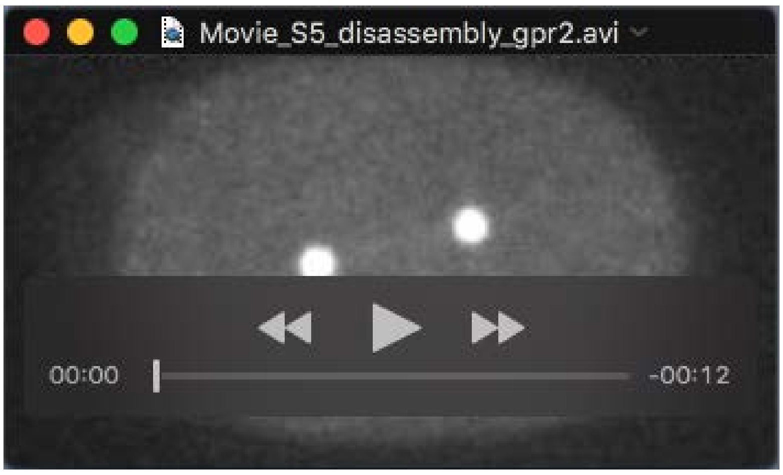

Movie S5. PCM disassembly in a *gpr-1/2(RNAi)* embryo expressing GFP::SPD-5.

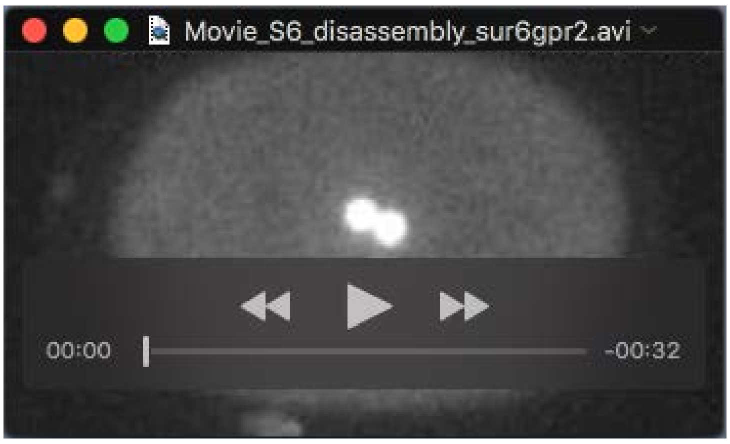

Movie S6. PCM disassembly in a *sur-6 gpr-1/2(RNAi)* embryo expressing GFP::SPD-5.
